# White Lupin Genomic Selection for Adaptation to Drought or Moderately Calcareous Soil: A Proof-of-Concept Study

**DOI:** 10.3390/ijms27094057

**Published:** 2026-04-30

**Authors:** Paolo Annicchiarico, Nelson Nazzicari, Luciano Pecetti, Tommaso Notario, Barbara Ferrari, Nicolò Franguelli, Daniele Cavalli

**Affiliations:** Research Centre for Animal Production and Aquaculture, Council for Agricultural Research and Economics, 26900 Lodi, Italy; nelson.nazzicari@crea.gov.it (N.N.); luciano.pecetti@crea.gov.it (L.P.); tommaso.notario@crea.gov.it (T.N.); barbara.ferrari@crea.gov.it (B.F.); nicolo.franguelli@crea.gov.it (N.F.)

**Keywords:** drought resistance, genotype × environment interaction, genomic prediction, grain yield, lime tolerance, *Lupinus albus*, plant-soil interaction

## Abstract

Genomic selection (GS) may improve the adaptation of white lupin to drought or moderately calcareous soil, enabling to realize its potential as a high-protein crop. This study aimed to (a) verify breeders’ ability to identify the top-, mid-, and bottom-performing genotypes of published GS models of breeding lines and landrace genotypes for adaptation to drought and moderately calcareous soil; and (b) compare the top-performing materials produced by GS and phenotypic selection. Twelve selected genotypes were evaluated in four managed environments obtained through combining two soils (non-calcareous; moderately calcareous) with two water treatments (moderate terminal drought; moisture-favorable). GS based on the genotyping-by-sequencing of independent material was challenged by validation conditions that were partly different from the training ones and an imposed similarity of genomically predicted genotype phenology (to exploit drought resistance rather than drought escape). Grain yield reduction relative to favorable conditions averaged 19% for drought and 23% for calcareous soil. GS correctly identified the top-performing material for drought-prone or moderately calcareous soil, except for one model based on a small training set. The best GS lines performed comparably to the best phenotypically selected material. A higher harvest index was associated with better adaptation to drought and calcareous soil. Crossover genotype × water treatment interaction underpinned the selection for adaptation to drought.

## 1. Introduction

White lupin (*Lupinus albus* L.) is a cool-season grain legume the seed of which is of increasing interest in the context of plant-based foods due to its high (34–45%) protein content and good amount of essential amino acids, as well as its several useful techno-functional properties, and the positive effects that it can exert on human health [1,2,3,4]. In addition, white lupin has high potential interest as a rain-fed high-protein crop for animal feed [5] and for aquaculture [6]. White lupin could be particularly important for organic and other low-input systems because of its good nitrogen fixing ability [7] and its ability to mobilize phosphorous from the soil [8].

White lupin tended to display a higher protein yield per unit area than other cool-season grain legumes in several cropping regions [9]. However, the expansion of this crop is generally limited by its economically insufficient yield [10], particularly in soils that are even mildly calcareous, i.e., those with active lime content above 1% [11], or in soils with an alkaline reaction [12]. Various studies suggest that active lime has a greater depressive effect on crop yield of *Lupinus* species than does alkalinity [13,14,15], but these factors are associated in calcareous soils. In Southern Europe and other Mediterranean-climate areas, white lupin yield also depends crucially on the crop’s ability to withstand drought, a stress that occurs alongside critical reproductive stages. The changing climate is expected to increase the presence and the severity of this stress in Europe [16] and elsewhere, e.g., in South America [17].

Plant breeding represents the main way to improve the adaptation of white lupin to rain-fed, drought-prone areas and moderately calcareous soils. Useful landrace genetic resources for both traits were identified. Several landrace accessions demonstrated higher yields relative to a set of modern varieties in the evaluation of a global germplasm collection in a moderately dry Mediterranean environment of Sardinia [18]. Remarkable genotype × environment interaction (GEI) for grain yield across drought and moisture-favorable managed environments indicated specific adaptation to severe drought by landraces with a different geographic origin and similar phenology [19]. Variation in adaptation to drought was modest in germplasm from Portugal [20]. It was sizeable among Egyptian landraces [21], which, however, were reportedly less adapted than the material from Italy [22]. Drought resistance, as indicated by phenology-independent adaptation to drought, is particularly important, because drought stress escape achieved by an early flowering (which depends on low vernalization requirements) can expose the crop to greater winter mortality in autumn-sown environments [23,24]. Variation in drought resistance was found in a large collection of breeding lines originating from crosses of elite landrace accessions with elite modern germplasms [25]. Adaptation to moderately calcareous soil, as indicated by relatively lower yield depression in unfavorable soil, was found in landrace accessions from Egypt [26,27] and Italy [28]. Variation in adaptation to calcareous soil also emerged within a large collection of breeding lines [29].

White lupin breeding for adaptation to drought or moderately calcareous soils is constrained by budget limitations and intrinsic difficulties. Field-based selection for drought resistance is limited by increasingly erratic climatic conditions, emphasizing the interest in the adoption of managed environments and large phenotyping platforms that allow for the extent of stress to be controlled [25]. Selection for lime tolerance may be complicated by high levels of soil spatial heterogeneity when performed under field conditions [30,31], and by abnormal plant root growth [32] and fairly modest correlation with genotype responses in agricultural environments when performed in pots or under hydroponic conditions [14]. Genomic selection (GS) may provide a cost-efficient solution for these traits and other ones that are genetically complex and difficult or expensive to evaluate. GS combines the phenotyping and genotyping data of a genotype sample representing a reference population to establish a statistical model for the prediction of breeding values in future plant selections [33,34]. This breeding strategy became practically feasible after the development of next-generation sequencing techniques, such as genotyping-by-sequencing (GBS) [35], that allow for large germplasm sets to be genotyped by thousands of single-nucleotide polymorphism (SNP) markers at a relatively low cost. The genome-enabled predictions developed for landrace germplasms could also be useful in identifying promising genetic resources in large grain legume germplasm collections [36,37] that cannot be thoroughly phenotyped due to limited budgets. The number of white lupin accessions exceeds 6300, even when considering only the main white lupin collections [38].

Prior studies have defined the genome-enabled prediction models for grain yield under managed severe drought for two white lupin reference populations, one including a genetically broad collection of sweet-seed breeding lines [25] and the other relative to a global landrace collection [39]. These models featured predictive ability values (as the Pearson correlation between predicted and observed values assessed by intra-environment cross validation) of 0.67 and 0.58, respectively. For the breeding lines, additional GS models were developed to predict the onset of flowering and phenology-independent yield under drought (termed ‘adjusted yield’ and computed as the genotype deviation from the yield value expected according to a linear regression of genotype yields as a function of onset of flowering) [25].

Genomic prediction models for the reference population of breeding lines were also developed for adaptation to a moderately calcareous soil in Greece as estimated from grain yield and a visual lime-susceptibility score [29]. The predictive ability of these models was 0.34. This study also included a second evaluation site, in the Netherlands, featuring lower soil lime content, modest genetic correlation with the Greek site for the genotype yield responses, and lower predictive ability of the site-specific GS model for yield (just 0.23).

There is increasing emphasis on the development of genome-enabled predictions for key traits for virtually all crops. Predictions have also been developed in white lupin for grain yield in subcontinental-climate or oceanic-climate conditions [39] and tolerance to anthracnose [40], frost resistance [41], and various seed quality traits [40,42]. However, the verification of the ability of these models to identify superior genotypes when applied to independent germplasm of the same reference population tested in independent environments is of paramount practical importance and is largely pending verification.

The main objective of this study was to verify the ability of three genomic prediction models to identify material with superior breeding value. Two of these models were relative to yielding ability under drought conditions, in breeding lines [25] and landrace genotypes [39], respectively; and one was relative to the yielding ability of breeding lines in a moderately calcareous soil [29]. The study was carried out on one site using four managed environments obtained through the factorial combination of two soil types (sandy-loam, non-calcareous; silty-clay, moderately calcareous) with two water treatments (moderate terminal drought; moisture-favorable conditions), thereby verifying the selection for each target trait across different environments (different soils, for adaptation to drought; different water amounts, for adaptation to moderately calcareous soil). Besides including genomically selected material, the study considered germplasm that was phenotypically selected within the experiments used for the construction of genomic prediction models, using this germplasm as a reference for a preliminary comparison of phenotypic *versus* genomic selection.

## 2. Results

**Figure 1:**
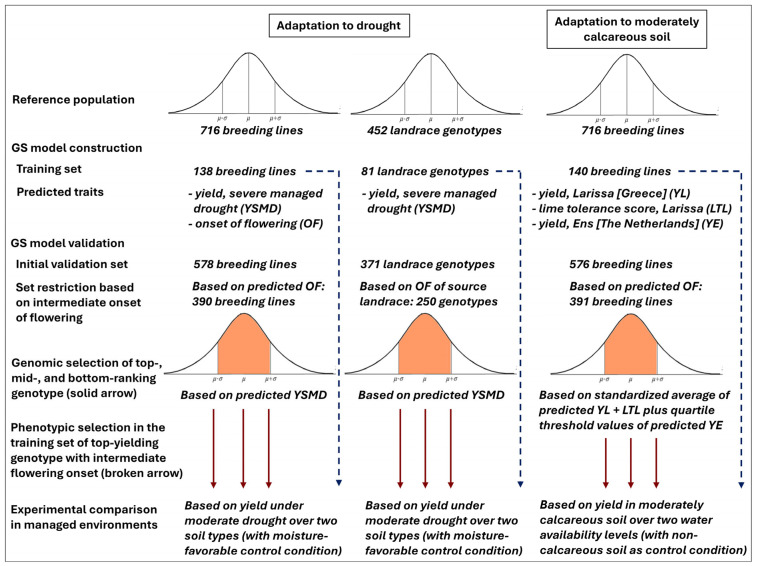
Schematic representation of the construction of genomic selection (GS) models for adaptation to drought in breeding line and landrace accession reference populations and adaptation to moderately calcareous soil in breeding lines, and their subsequent validation in four managed environments. For each target trait and reference population, the validation included the top-yielding genotype from the training set and the predicted top-, mid-, and bottom-performing genotypes from an independent validation set, including in all cases only material with actual or predicted intermediate phenology.

The evaluation in the four managed environments included 12 white lupin genotypes that belonged to three distinct groups of material with respect to the proof-of-concept study: (a) breeding lines selected for adaptation to drought; (b) landrace genotypes selected for adaptation to drought; (c) breeding lines selected for adaptation to moderately calcareous soil. Each group included four genotypes, of which one was selected phenotypically as top-yielding within the training set used in the phenotyping experiment for GS model construction, and three were selected as predicted top-, mid-, and bottom-yielding according to genome-enabled predictions applied to other (independent) genotypes of each reference population (Figure 1). Each group of genomically selected genotypes had to satisfy the condition of modest variation for onset of flowering (based on genomic predictions for breeding lines and on data associated with the source accessions for landrace genotypes), to limit the overwhelming impact of a very early-flowering phenology on adaptation to drought (Figure 1).

There were some differences between the phenotyping environments used for GS model construction and those used for GS validation. Compared with the drought stress imposed on managed environments for GS model construction, the stress adopted for validation of adaptation to drought of breeding lines and landrace genotypes was (a) more terminal because of the more advanced growth stage of the plants at stress application (as a result of an earlier sowing), and (b) less severe because of the much greater amount of water provided from previous autumn/early-winter rainfall (Table 1). The validation conditions, which were closer to those actually occurring in the Mediterranean and sub-continental climate agricultural sites of Italy, implied a smaller reduction in mean grain yield when going from moisture-favorable to drought stress treatments compared with the yield reduction observed in the training environments, particularly for the landrace reference population (Table 1). Some difference between GS model training and GS validation environments occurred also for adaptation to moderately calcareous soil, as the soils of the agricultural environments of Larissa (Greece) and Ens (The Netherlands) that were used for the construction of the main and secondary GS models, respectively, contained less total and active lime than the silty-clay soil of the managed environment used for GS validation (Table 2). The differences between the silty-clay and sandy-loam soils of the validation environments in terms of amounts of irrigation water and yield reductions due to drought stress, as described in Table 1, were essentially due to the greater water-holding capacity of the silty-clay soil and the consequently greater amount of water that was needed to reach benchmark levels of soil water available for this soil.

In an analysis of variance (ANOVA) performed for dry grain yield and six other traits (dry straw biomass, harvest index, flowering time, maturity time, plant height, and individual seed weight) of all genotypes, significant (*p* < 0.05) variation was found (a) among genotypes across environments, for all traits; (b) among environments, for all traits except seed weight; (c) in GEI due to water amount, for grain yield, straw biomass, harvest index, and seed weight; (d) in GEI due to soil type, for all traits; (e) in genotype × water amount × soil type interaction, for grain yield, straw biomass, harvest index, and maturity time (Supplementary Table S1). The ‘drought stress/silty-clay soil’ environment, which combined drought stress and moderately calcareous soil, showed the lowest mean grain yield; in contrast, the ‘moisture-favorable/sandy-loam soil’ environment was the top-yielding one (Table 3). Based on the environment mean yields reported in Table 3, there was a grain yield reduction of 19% when going from moisture-favorable to drought stress conditions, as averaged across soil types, and a yield reduction of 23% in going from non-calcareous to moderately calcareous soil, as averaged across water treatments. The differences in environment mean for straw biomass tended to parallel those for grain yield, but with somewhat greater stress-induced reduction (29% due to drought stress across soil types; 27% due to unfavorable soil across water treatments; Table 3). In the most stressful environment (combining drought and unfavorable soil), the grain yield penalty was alleviated by a higher harvest index than found in any other environment (Table 3). Plants matured earlier in drought-stressed than in moisture-favorable environments and, within the same water treatment, matured earlier in the favorable soil than in the moderately calcareous one (Table 3).

The GS models under validation were constructed for different traits (adaptation to drought or calcareous soil) and reference populations (breeding lines or landrace genotypes), and were applied to different sets of genotyped plants (Figure 1). Therefore, the comparison of predicted contrasting genotypes (top-, mid-, or bottom-performing ones) based on GS, and that of top-yielding material issued from GS and phenotypic selection, were limited to the four genotypes representing each target trait and reference population. The results of separate ANOVAs performed for each of these sets of genotypes (breeding lines selected for adaptation to drought; landrace genotypes selected for adaptation to drought; breeding lines selected for adaptation to moderately calcareous soil) are summarized in Supplementary Table S2. Genotype comparisons within each group are subsequently reported: (a) separately for drought stress and moisture-favorable conditions, for validation of GS models for adaptation to drought in the presence of significant (*p* < 0.05) genotype × water amount interaction; (b) separately for moderately calcareous and non-calcareous soil types, for validation of GS models for adaptation to calcareous soil in the presence of significant (*p* < 0.05) genotype × soil type interaction; and (c) averaged across conditions, in the absence of relevant interactions.

The results for the breeding lines selected for adaptation to drought are given in Table 4. The GS model predicted correctly the breeding values of new lines, since those predicted as top-, mid- and bottom-yielding ranked in this order experimentally and differed significantly (*p* < 0.05) for grain yield under drought stress. The phenotypically selected genotype displayed a slight, non-significant yield disadvantage under drought relative to the genomically predicted top-yielding genotype. Interestingly, the yield responses involved significant (*p* < 0.05) crossover interactions across water treatments with respect to the predicted top-yielding and mid-yielding genotypes, since the former out-yielded the latter under drought while being out-yielded in the moisture-favorable environment. The yield advantage under drought of the predicted top-yielding genotype over the mid-yielding one was associated with higher harvest index in the presence of similar straw biomass, earlier onset of flowering, somewhat earlier maturity, a shorter plant, and smaller seeds. However, the difference in flowering time between the predicted top-, mid-, and bottom-performing genotypes was modest (3.4 days), as expected from the imposed condition of similarity of genomically predicted onset of flowering. Higher harvest index and earlier phenology were more pronounced in the phenotypically selected genotype.

The results for the landrace genotypes selected for adaptation to drought are given in Table 5. In this case, the genomic predictions were partly unsuccessful in distinguishing the breeding values for grain yield under drought, since the predicted mid-yielding genotype out-performed the bottom-yielding genotype (*p* < 0.05) but showed no significant difference from the predicted top-yielding one. The phenotypically selected genotype had a yield comparable with those of the GS-predicted mid- and top-yielding genotypes under drought, while showing higher yield under moisture-favorable conditions. This widely adapted genotype relied on a consistently higher harvest index and earlier-flowering phenology relative to the other genotypes in drought stress and moisture-favorable environments. The GS-identified genotypes exhibited modest phenological differences according with expectations, along with a trend towards a higher straw biomass for the predicted top-yielding genotype and towards a higher harvest index for the predicted mid-yielding one.

Table 6 reports the results for the breeding lines selected for adaptation to moderately calcareous soil. In this case, GS successfully predicted the top-yielding genotype in the moderately calcareous soil, whereas the predicted mid- and bottom-yielding genotypes ranked in this order but did not differ at *p* < 0.05 for yield in this soil. The yielding ability of the phenotypically selected genotype in the calcareous soil was slightly lower than that of the GS-predicted top-yielding genotype and did not differ from any genotype at *p* < 0.05. The genotype × soil type interaction for grain yield was mainly characterized by the much better response in the non-calcareous soil of the genotype predicted as mid-yielding in the calcareous soil. The genotypes had a small range of variation for onset of flowering (three days) also in this case. The specific adaptation to calcareous soil of the GS-predicted top-yielding line was associated with a high harvest index and a slightly later flowering (rather than slight flowering anticipation) in this condition relative to the values in the non-calcareous soil, along with a somewhat earlier maturity and a larger seed, consistently across soil types.

## 3. Discussion

The current use of managed environments offered the advantage of assessing crop and genotype responses to moderate terminal drought stress and moderately calcareous soil with a full control of water amounts and in the absence of differences for other climate or crop management factors (e.g., temperatures; diseases). This would not be possible in experiments performed across agricultural locations that contrast for drought stress or type of soil—in which genotype responses to water amounts or soil types could be confounded with those due to other environmental factors. We adopted a late-winter sowing because an autumn sowing may have introduced a bias in the assessment of adaptive responses due to genotype variation in resistance to low winter temperatures, a variation which is large within the reference population of breeding lines [41] and the accessions from which the landrace genotypes were selected [18]. As anticipated, the adopted validation conditions implied an intensity and a pattern of drought stress that were closer to those of our target environments than those used for GS model construction. Validation conditions were somewhat different from those used for GS model construction also with respect to the active lime content of the moderately calcareous soil, which was currently higher (Table 2). Active lime affects *Lupinus* species directly [43], and even more so indirectly, through the precipitation of organic acids secreted by lupin cluster roots to mobilize and effect the uptake of phosphorus and iron [44] and through the inhibition of iron uptake by HCO_3_^−^ [45]. While the control of environmental factors enabled by the managed environments reduced the need for a repetition in time of the evaluation, the use of these environments necessarily constrained the number of genotypes that could be evaluated. The small number of predicted contrasting lines that could be selected for each GS model represented a major limitation of this proof-of-concept study.

The stress conditions due to drought or unfavorable soil exerted a similar grain yield reduction relative to favorable conditions (19% for moderate drought; 23% for moderately calcareous soil). On average, the drought stress led to an anticipation of physiological maturity, a well-known and general plant plasticity response aimed at assisting the crop stress escape [46]. In contrast, plants grown in the calcareous soil delayed their maturity in comparison with the favorable soil—a response already observed in earlier work in managed environments [28], albeit difficult to explain. In the ‘drought stress/silty-clay soil’ environment, a partial compensation for the negative effects on plant production exerted by the combination of the two stresses was represented by an increase in harvest index. Higher harvest index has previously emerged as a white lupin response to a moderately calcareous soil in an earlier study in managed environments [28]. It has also occurred in response to increasingly drought-stressed conditions, such as those used for GS model training of the breeding lines [25], but not under the even more stressing conditions used for GS model training of the landrace genotypes [39].

Our validation conditions were different from those used for the definition of GS models, not only as to the extent and pattern of drought and the level of soil active lime, but also because the validation assessed the genotypes’ adaptation to moderately calcareous soil across different water amounts (drought stress or moisture-favorable) and the genotypes’ adaptation to drought stress across different soil types (sandy-loam or silty-clay). These circumstances made genome-enabled predictions more challenging, but also more realistic for breeders, who would exploit these predictions for applications in newly drought-prone or moderately calcareous target environments that are necessarily subjected to environmental variation and are somewhat different from the training environment used for GS. Genome-enabled selection was further challenged by imposing the condition that the set of predicted top-, mid-, and bottom-performing lines should possess a similar phenology. This criterion reduced substantially the genetic variation for adaptation to drought, which would otherwise depend markedly on drought escape by an early phenology [19,25,47]. As anticipated, focusing on drought resistance rather than drought escape has strategic importance for autumn-sown white lupin in the target region, as an early-flowering phenology also implies greater susceptibility to low winter temperatures [18,24].

The validation of a GS model for the prediction of onset of flowering for the breeding lines was not a specific objective of this study. However, the model that we used for pre-selection of the breeding lines with predicted similar onset of flowering was successful, since the maximum observed range of variation in the onset of flowering (<4 days) was modest and consistent with expectations for the specific set of selected lines (<5 days). The GS model for the onset of flowering had shown a high predictive ability (0.76) based on intra-environment cross validations [25], in agreement with the substantial genetic control of this trait by major genes in white lupin [48] and the generally good ability to predict flowering time in crop plants [49].

The GS model for the prediction of grain yield under drought stress was fully satisfactory for the reference population of breeding lines while being only partly satisfactory for the reference population of landrace genotypes. Three reasons may have converged to prompt less-accurate predictions for the landrace genotypes relative to the breeding lines: (a) the greater difference between phenotyping and validation conditions with respect to the landrace germplasm, owing to the particularly low water amount under drought adopted for this material during phenotyping aimed to GS model construction (Table 1); (b) the somewhat lower predictive ability of the GS model for the landrace germplasm relative to the breeding lines, according to intra-environment cross validations (0.58 vs. 0.67) [25,39]; and (c) the smaller training population size used for constructing the GS model for the landrace material relative to the breeding lines (81 vs. 138 genotypes). The last element was important, as a large training size is needed for a satisfactory prediction of a trait, such as crop yield, that is genetically complex and has only moderate heritability. For example, the predictive ability of soybean yield in a nested association mapping panel showed a sharp increase passing from 50 to 100 genotypes as the training population size, with progressively smaller rates of increase observed until it reached a plateau at 250 genotypes [50]. The predictive ability of grain yield in a global pea germplasm collection increased until it reached a plateau at 175 genotypes [37]. A sharp rise in predictive ability was observed when going from 90 to 120 training genotypes (with smaller rates of increase up to 200 genotypes) for two component traits of seed yield in another pea germplasm collection [51]. Combining the distinct GS models set up for breeding lines and landrace accessions into a unique model was inconvenient, because of the different reference populations and the somewhat different phenotyping conditions. Earlier work on white lupin frost resistance and grain quality traits showed a distinct drop in predictive ability when passing from intra-population to inter-population predictions, especially when using a model constructed for breeding lines to predict landrace material [41,42]. A similar result was reported also for another inbred crop, namely, wheat [52].

The occurrence of significant (*p* < 0.05) crossover interaction across water treatments of the predicted top- and mid-yielding lines emphasized the importance of selection for specific adaptation to drought in white lupin. A crossover interaction for grain yield across drought stress and favorable conditions was already observed in landrace accessions featuring similar phenology [19].

Genome-enabled predictions for breeding line adaptation to moderately calcareous soil were moderately successful, as they were able to correctly distinguish well-adapted material from less-adapted ones. A lower accuracy of GS for this trait relative to adaptation to drought of the breeding lines was expected on the basis of the lower predictive ability of its component traits according to intra-environment cross validations (0.34 for both grain yield and a visual lime susceptibility score in Greece vs. 0.67 for grain yield under drought) in the presence of similar training population size (140 vs. 138 genotypes) [25,29].

Our study only allowed for a preliminary comparison of phenotypically vs. genomically selected lines extracted from the same reference population. However, our results are encouraging for GS aimed at adaptation to drought or moderately calcareous soil, as the predicted top-yielding breeding line according to GS yielded comparably (actually, with a slight, non-significant yield advantage) to the top-performing line of the training set used for the GS construction, for both target traits. This is remarkable when considering the lower cost and duration of one selection cycle for GS relative to phenotypic selection.

The small number of evaluated lines provided only incidental information on relevant adaptive traits. Higher harvest index was associated with the adaptation of better-performing material to drought or to calcareous soil. For adaptation to drought, this result agreed with earlier findings relative to a large set of genotypes [25]. The larger seed featured by the top-yielding line in the calcareous soil would agree with results associated with a larger set of genotypes [28], given the possible advantage in an unfavorable soil of the greater early shoot and root growth that are associated with a larger seed [23].

In conclusion, this proof-of-concept study indicated that GS, when based on a sufficient training population size, could conveniently be used to select white lupin genotypes adapted to drought-prone environments or moderately calcareous soil. The GS of breeding lines for drought conditions exploited mainly the variation for drought resistance, thanks to an additional genomic prediction model able to predict onset of flowering. The practical importance of selection for adaptation to drought was reinforced by the occurrence of GEI of a crossover type between selected and unselected lines, passing from stress to moisture-favorable conditions. The performance of the predicted top-performing lines according to GS was comparable to that of the best phenotypically selected material. While being meaningful for the purpose of the study, the currently small number of test genotypes could hardly be used for improving the predictive ability of the GS models. Such an improvement, which is particularly important for adaptation to drought of landrace accessions based on our results, ought to rely on additional model training work. An example of improved GS predictions arising from additional model training work was recently provided by Patyi et al. [53] for white lupin tolerance to anthracnose.

## 4. Materials and Methods

### 4.1. Reference and Training Populations

Our study included two white lupin reference populations, one relative to breeding lines and the other relative to landrace genotypes (Figure 1). The former consisted of 716 lines originating from 16 crosses produced through a 4 × 4 factorial mating design. Each of four elite, sweet-seed cultivars or breeding lines, namely, the French cultivar Lucky, the French breeding line MB-38, the Italian variety Arsenio, and the Moroccan breeding line L27PS3, was crossed with each of four elite, bitter-seed landrace accessions, namely, the Italian landraces LAP123 and La246, the landrace La646 from the Canary Islands, and the Greek landrace Gr56. The selection of the parent germplasm, which was based on desirable agronomical traits, and the development of the inbred lines until F_6_ seed under insect-proof cages (to prevent cross-pollinations), were described previously [25]. The lines underwent a selection for low alkaloid content in F_3_ and F_4_ generations. Each parent genotype contributed to the reference population with a similar number of lines. Phenotyping for grain yield under drought was performed on a training set of 138 lines in a managed stress environment in Lodi (Italy) under the conditions summarized in Table 1 and thoroughly described in [25]. Phenotyping for grain yield in moderately calcareous soil was carried out under field conditions, as reported in [29] on a training set of 140 lines in two locations, namely, Larissa (Greece) and Ens (The Netherlands), on soils the main characteristics of which are reported in Table 2. The training sets for adaptation to drought and adaptation to calcareous soil had most lines in common and featured a moderately balanced number of lines issued from each of the eight parent lines [25,29]. The selection of contrasting lines was based on genome-enabled predictions applied to independent lines that were excluded from the training sets used for the GS model construction (i.e., 578 and 576 lines for adaptation to drought and moderately calcareous soil, respectively).

The reference population of landrace genotypes was developed from the 113 landrace accessions described in [18], which belonged to 11 regional germplasm pools reflecting the main historical white lupin cropping regions. Each region was represented by at least eight accessions. Four genotypes were randomly extracted from each accession, producing a reference population of 452 genotypes. The genotypes selected from the same landrace accession were genetically distinct, as confirmed by the observed molecular marker diversity and consistently with the expected intra-landrace variation. Phenotyping for grain yield under drought was performed on a training set of 81 genotypes in a managed stress environment in Lodi (Italy) under the conditions summarized in Table 1 and described in [39]. The training set included 78 genotypes sorted out of the landrace reference population, as well as three genotypes that acted as sweet-seed parent genotypes of the reference population of breeding lines. The selection of the contrasting lines for this study was based on genome-enabled predictions applied to 371 landrace genotypes that were excluded from the training set.

### 4.2. Molecular Characterization

The entire set of genotypes belonging to each reference population underwent GBS characterization through the steps of DNA isolation, GBS library construction, sequencing, SNP calling, and data filtering that were described in earlier reports relative to the phenotyping work [25,29,39]. In brief, genomic DNA extracted from young leaves of landrace genotypes and F_5_ plants of breeding lines was sent to The Elshire Group Ltd. laboratory (Palmerston North, New Zealand) for outsourced library preparation and sequencing. GBS data were generated according to [35] using the *ApeK*I restriction enzyme (NEB New England Biolabs, R0643L, Ipswich, MA, USA) and amplifying the library by Kapa Taq polymerase Alpha (KAPA Library Amplification Readymix, Kapa Biosystems KK2611, Cape Town, South Africa). The adoption of *ApeK*I was supported by the fact that about 60% of the white lupin genome includes repetitive DNA sequences [54], which this enzyme tends to skip.

The SNP calling was based on the Legpipe2 pipeline default settings for diploid species [55]. For alignment, we used the *Lupinus albus* genome version 1.0 [54], which was downloaded from https://www.whitelupin.fr/ (accessed on 8 September 2025). The datasets of the two reference populations were filtered independently for monomorphic markers: missing rate per marker < 0.3, missing rate per genotype < 0.5, and SNP heterozygosity < 0.3. This process retained 32,951 SNPs for breeding lines and 40,914 SNPs for landrace genotypes. We used random forest imputation to estimate missing data [56], using the R 4.5.2 package MissForest [57] with the configuration ntree = 100, maxiter = 10 and encoding genotypes as categorical data (factors).

### 4.3. Genomic and Phenotypic Selections

A schematic representation of the definition of genomically and phenotypically selected material for the proof-of-concept study is reported in Figure 1. The selection of the predicted top-, mid-, and bottom-yielding genotypes was based on the application of GS models reported in earlier studies. Predicted contrasting breeding lines for grain yield under drought were identified according to the average values of line breeding values predicted by two statistical models, namely, Ridge Regression BLUP (rrBLUP) and Weighted G-BLUP (WGBLUP), which showed similar predictive ability based on intra-environment cross validations in the phenotyping experiment [25]. Predictions based on the latter statistical model were also generated for onset of flowering, as reported in [25], to impose the additional condition of similar onset of flowering of the selected genotypes. We selected the predicted top-yielding, bottom-yielding, and mid-yielding genotypes (where mid-yielding meant the greatest similarity with the mean of the predicted breeding values) among materials whose flowering time was comprised in the range of *m* ± *s*, where *m* and *s* stand for the mean and the standard deviation, respectively, of the predicted onset-of-flowering values for the genotypes. About 67.5% of the lines (390 out 578) met this phenology requirement and were candidates for selection. The selected genotypes had a predicted range of onset of flowering slightly smaller than five days. Incidentally, we verified that the selected material would also have been selected through the GS model for prediction of phenology-independent yield under drought, termed ‘adjusted yield’ in [25].

Predicted contrasting landrace genotypes for grain yield under drought were identified according to breeding values predicted by rrBLUP, which had displayed high intra-environment predictive ability based on the phenotyping data [39]. In this case, the condition of similar onset of flowering for the predicted contrasting genotypes was imposed by restricting the selection to genotypes for which the source landrace accession had showed onset of flowering within the range of *m* ± *s* consistently in each of four evaluation environments represented by three European locations in [18] and the one greenhouse environment in [58]. About 67.3% of the accessions (76 out of 113) met this requirement, restricting the candidate genotypes for selection to about 250.

The identification of predicted contrasting breeding lines for adaptation to moderately calcareous soil relied on a more complex GS procedure, compared with the adaptation to drought. Data from Larissa, which featured somewhat more calcareous soil (Table 2) and greater climate similarity to Italy than the site of Ens, were used to define the main GS models used for selection. Breeding values of grain yield and a visual lime susceptibility score based on data from this site were genomically predicted by rrBLUP and were standardized to zero mean and unit standard deviation; the susceptibility score was transformed into a tolerance score by inverting its sign; and an overall breeding value for adaptation to moderately calcareous soil was obtained by averaging the standardized values of the two variables. Also here, candidate genotypes for selection had to meet the additional criterion of genomically predicted onset of flowering (assessed as above) in the range of *m* ± *s*. One additional selection criterion relied on a secondary rrBLUP-based GS model relative to grain yield in Ens. We selected as the top-yielding line the first one according to standardized GS-predicted data in Larissa that also had a suitable GS-predicted phenology and for which the GS-predicted yield in Ens was within the 25% top quartile. Likewise, the selected bottom-yielding line was the bottom-yielding genotype according to predicted data in Larissa, had a suitable predicted phenology, and had predicted yield in Ens below the average. The selected mid-yielding genotype was predicted to be mid-performing in Larissa, showed suitable phenology, and fell within the second or third quartile for predicted yield data in Ens.

The breeding line that was phenotypically selected for adaptation to drought was the top-yielding line of the training set evaluated in [25] that concurrently satisfied the condition of onset of flowering in the range of *m* ± *s* based on phenology data observed in the same experiment. The landrace genotype phenotypically selected for adaptation to drought was the top-yielding genotype of the training set in [39] for which the source landrace accession satisfied the condition of onset of flowering in the range of *m* ± *s* in each of four agricultural or greenhouse evaluation environments [18,58]. Finally, the breeding line phenotypically selected for adaptation to moderately calcareous soil was a line featuring outstanding grain yield, both in Larissa (where it was second-ranking) and in Ens (where it was top-ranking), along with onset of flowering within the range of *m* ± *s* in both phenotyping experiments.

### 4.4. Proof-of-Concept Experiment

The experiment was carried in Lodi (45°18′10″ N; 9°30′43″ E), Northern Italy, in four managed environments defined through the factorial combination of two soil types (sandy-loam, non-calcareous; silty-clay, moderately calcareous) and two water treatments (moderate terminal drought; moisture-favorable). Each environment was represented by a large (24.0 m × 1.6 m × 0.8 m deep), bottomless container made of concrete, which was filled with one of the two soil types and equipped with a drip-irrigation system. The four environments, set close to each other, were placed under a transparent, moving, rain-out shelter. The total and active lime and pH of the two soil types are reported in Table 2. The sandy-loam soil had the following additional characteristics: 56% sand, 32% silt, and 12% clay; organic matter content = 1.4%; C/N = 9.0; volumetric field capacity of 20%; and a wilting point of 8%. The silty clay soil had 8% sand, 54% silt, and 38% clay; organic matter content = 4.6%; C/N = 9.2; volumetric field capacity of 36%; and a wilting point of 23%.

The rain-out shelter was open for most of the three-month period preceding the sowing, which took place on 21 January 2025. Rainfall over this period amounted to 220 mm, which represented well the long-term amounts during the same period associated with most locations in the Po Valley and in Central Italy [59]. The shelter was closed from sowing onwards. The water treatments were imposed starting from 17 April, when most genotypes had started to flower. In the moisture-favorable treatment, irrigation was provided when the available soil water (as defined by the difference in water content between field capacity and wilting point) in the upper 40 cm layer decreased below 60%, bringing it back to 80%. No irrigation was contemplated for the drought stress treatment. A moisture-favorable regime was applied throughout the period from 21 January to 17 April. A Diviner 2000 capacitance sensor (Sentek Pty Ltd., Stepney, Australia) monitored the soil moisture every three days. The water amounts received by each soil type before and after water treatment imposition are summarized in Table 1.

Within each environment, the genotypes were arranged according to a randomized complete block design with four replications. Each plot was 0.90 m large and 0.80 m long. It consisted of six rows spaced 0.15 m, with four plants per row spaced 0.20 m (plant density: 33 plants/m^2^). A mineral fertilizer incorporated into the soil provided 27 kg/ha of N, 46 kg/ha of P_2_O_5_, and 50 kg/ha of K_2_O. The seed was inoculated with Vitalianz R Lupin inoculant (Cérience, Cissé, France) prior to sowing. Weeds were controlled by hand removal.

Phenotypic data were collected on a plot basis excluding the plant row along the border of the container to avoid border effects. Grain yield and the biomass of straw (the latter also including the legume pod walls, in addition to shoots and leaf residues) were recorded after hand-threshing and drying at 60 °C until constant weight. In addition, we recorded onset of flowering (when 50% of the plants had one open flower) and physiological maturity time in days from 1 April; plant height at maturity (by averaging the data of four plants per plot relative to the distance between the soil and the tip of the highest shoot); harvest index (computed from grain and straw dry weight data); and individual seed weight (as average dry weight of a subsample of 100 seeds per plot).

### 4.5. Data Analysis

An ANOVA including the fixed factors genotype and environment and the random factor block within environment was performed for each trait of the whole set of genotypes, using block within environment as error term for environment (Supplementary Table S1) in accord with [60]. A second ANOVA including the fixed factors genotype, water amount and soil type and the random factor block within environment (as water amount × soil type combination) partitioned the variation of the environment factor and its interaction with genotypes into source of variation relative to water amount, soil type, and water amount × soil type interaction (Supplementary Table S1). The latter ANOVA was performed separately for traits of the three sets of genotypes (breeding lines selected for adaptation to drought; landrace genotypes selected for adaptation to drought; and breeding lines selected for adaptation to moderately calcareous soil), comparing the four genotypes of each group according to Least Significant Difference (LSD) values. Depending on results summarized in Supplementary Table S2, the comparisons referred to: (a) drought stress and moisture-favorable conditions, for validation of GS models for adaptation to drought in the presence of significant (*p* < 0.05) genotype × water amount interaction; (b) moderately calcareous and non-calcareous soil types, for validation of GS models for adaptation to calcareous soil in the presence of significant (*p* < 0.05) genotype × soil type interaction; (c) average values across conditions, in the absence of relevant interactions.

## Figures and Tables

**Table 1:** Water amount (WA, mm) for drought stress (DS) and moisture-favorable (MF) water treatments within three months before sowing (BS), from sowing to application in mid-April of water treatments (SA), and during application of water treatments, and reduction in total WA and mean grain yield of the stress treatment relative to the moisture-favorable one, for managed environments used for training and validation of genomic selection (GS) models for adaptation to drought of white lupin reference populations relative to breeding lines (BL) and landrace genotypes (LG).

Activity	Material	Soil Type	Sowing Time	WA, BS ^a^	WA, SA	WA, DS ^b^	WA, MF ^c^	WA Reduction ^d^ (%)	Yield Reduction (%)
GS training ^e^	BL	Sandy-loam	Mid-Feb.	30	120	50	240	49	61
GS training ^f^	LG	Sandy-loam	Mid-Feb.	30	120	21	332	64	78
GS validation	BL + LG	Sandy-loam	Mid-Jan.	220	80	0	70	19	15
GS validation	BL + LG	Silty-Clay	Mid-Jan.	220	100	0	200	38	24

^a^ WA provided by irrigation under a fixed rain-out shelter for GS model training environments and by rainfall in a mobile rain-out shelter for GS validation environments. ^b^ Irrigation when reaching 0% soil available water (SAW) to restore 15% SAW, for GS model training; absence of irrigation for GS validation. ^c^ Irrigation when reaching 60% soil available water (SAW) to restore 80% SAW, for both GS model training and validation. ^d^ Reduction in the total amount of water received in the stress treatment (BS + SA + DS) compared to that received in the moisture-favorable one (BS + SA + MF). ^e^ See details in [25]. ^f^ See details in [39].

**Table 2:** Soil characteristics and water amounts for (a) agricultural environments used for training of main and secondary genomic selection (GS) models for white lupin adaptation to moderately calcareous soil; (b) managed environments with moderately calcareous (silty-clay) soil, used for GS validation; and (c) managed environments with non-calcareous (sandy-loam) soil.

Environment	Use of the Environment	Total CaCO_3_ (g/kg)	Active CaCO_3_ (g/kg)	pH (in H_2_O)	Water over Crop Cycle (mm)
Larissa (Greece) ^a^	Training of main GS model	61	22	7.6	265
Ens (the Netherlands) ^a^	Training of secondary GS model	50	18	7.9	433
Managed, Silty-clay soil	GS validation	123	30	7.5	372 ^b^
Managed, Sandy-loam soil	Non-target environment	12	5	7.6	372 ^b^

^a^ See details in [29]. ^b^ Average value for the drought stress and moisture-favorable managed environments reported in Table 1.

**Table 3:** Mean comparison for six white lupin traits of four managed environments generated by the factorial combination of two water amounts (drought stress; moisture-favorable) and two soil types (sandy-loam, non-calcareous; silty-clay, moderately calcareous).

Environment	Grain Yield (t/ha)	Straw Biomass (t/ha)	Harvest Index	Flowering Time (dd from 1 April)	Maturity Time (dd from 1 April)	Plant Height at Maturity (cm)
Drought stress/Sandy-loam soil	3.67 b	4.33 b	0.456 b	15.7 b	76.8 d	102.5 a
Drought stress/Silty-clay soil	2.63 c	2.55 c	0.510 a	15.7 b	82.8 b	72.3 c
Moisture-favorable/Sandy-loam soil	4.31 a	5.19 a	0.449 b	16.1 ab	81.3 c	108.7 a
Moisture-favorable/Silty-clay soil	3.49 b	4.44 b	0.446 b	16.3 a	90.3 a	92.1 b
LSD (*p* < 0.05)	0.32	0.53	0.019	0.4	0.9	7.3

Means differ at *p* < 0.05 when followed by different letter(s).

**Table 4:** Mean comparison of white lupin breeding lines, of which one is top-yielding based on phenotypic selection (PS) and three are top-, mid- or bottom-yielding based on genomic selection (GS) for adaptation to drought, for grain yield (GY, t/ha), straw biomass (SB, t/ha), harvest index (HI), flowering time (FT, days from 1 April), maturity time (MT, days from 1 April), plant height at maturity (PH, cm), and individual seed weight (SW, g). Values are in drought stress (D) or moisture-favorable (F) managed environments, or mean values across environments (the latter reported in the absence of genotype × water amount interaction).

Genotype	GY, D	GY, F	SB, D	SB, F	HI, D	HI, F	MT, D	MT, F	FT, Mean	PH, Mean	SW, Mean
PS, top	3.45 ab	3.90 bc	3.39 b	4.48 b	0.515 a	0.468 a	79.3 b	86.3 cd	15.3 d	91.6 c	0.337 c
GS, top	3.75 a	4.25 b	3.94 a	4.76 b	0.500 a	0.473 a	80.0 b	85.9 d	16.5 c	90.2 c	0.329 c
GS, mid	3.19 b	4.96 a	4.01 a	6.50 a	0.447 c	0.432 b	81.6 a	87.0 bc	19.9 a	106.6 a	0.434 a
GS, bottom	2.66 c	3.66 c	3.13 b	4.88 b	0.466 b	0.429 b	82.1 a	89.0 a	17.9 b	99.8 b	0.364 b
LSD (*p* < 0.05)	0.45	0.55	0.48	0.68	0.017	0.019	1.0	1.0	0.6	3.5	0.013

Means averaged across two soil types; they differ at *p* < 0.05 when followed by different letter(s).

**Table 5:** Mean comparison of white lupin landrace genotypes, of which one is top-yielding based on phenotypic selection (PS) and three are top-, mid- or bottom-yielding based on genomic selection (GS) for adaptation to drought, for grain yield (GY, t/ha), straw biomass (SB, t/ha), harvest index (HI), flowering time (FT, days from 1 April), maturity time (MT, days from 1 April), plant height at maturity (PH, cm), and individual seed weight (SW, g). Values are in drought stress (D) or moisture-favorable (F) managed environments, or mean values across environments (the latter reported in the absence of genotype × water amount interaction).

Genotype	GY, D	GY, F	SB, D	SB, F	HI, D	HI, F	FT, Mean	MT, Mean	PH, Mean	SW, Mean
PS, top	3.65 ab	5.02 a	3.21 c	4.50 c	0.541 a	0.527 a	13.7 c	79.7 c	89.4 b	0.280 c
GS, top	3.54 ab	4.42 b	4.22 a	6.73 a	0.451 c	0.392 c	14.7 b	82.4 a	98.1 a	0.309 ab
GS, mid	3.83 a	4.64 b	3.77 ab	4.76 c	0.508 b	0.493 b	15.4 a	81.5 bc	97.2 a	0.322 a
GS, bottom	3.28 b	3.68 c	3.77 ab	5.66 b	0.468 c	0.393 c	15.5 a	82.0 ab	87.0 b	0.300 b
LSD (*p* < 0.05)	0.45	0.55	0.48	0.66	0.017	0.019	0.6	0.8	3.5	0.013

Means averaged across two soil types; they differ at *p* < 0.05 when followed by different letter(s).

**Table 6:** Mean comparison of white lupin breeding lines, of which one is top-yielding based on phenotypic selection (PS) and three are top-, mid- or bottom-yielding based on genomic selection (GS) for adaptation to moderately calcareous soil, for grain yield (GY, t/ha), straw biomass (SB, t/ha), harvest index (HI), flowering time (FT, days from 1 April), maturity time (MT, days from 1 April), plant height at maturity (PH, cm), and individual seed weight (SW, g). Values are in moderately calcareous (silty-clay, C) or non-calcareous (sandy-loam, NC) managed environments, or mean values across environments (the latter reported in the absence of genotype × soil type interaction).

Genotype	GY, C	GY, NC	SB, C	SB, NC	HI, C	HI, NC	FT, C	FT, NC	PH, C	PH, NC	MT, Mean	SW, Mean
PS, top	2.66 ab	2.82 b	2.85 a	4.03 bc	0.482 b	0.407 b	16.4 a	16.3 ab	83.1 a	106.9 a	83.5 b	0.315 c
GS, top	2.75 a	3.75 a	2.72 ab	4.55 ab	0.506 a	0.452 a	14.6 b	14.1 c	76.0 b	104.1 a	82.1 c	0.350 a
GS, mid	2.28 b	3.90 a	2.59 ab	4.78 a	0.474 b	0.450 a	14.9 b	15.8 b	74.0 b	107.8 a	84.0 b	0.334 b
GS, bottom	2.14 b	2.42 b	2.30 b	3.55 c	0.486 b	0.411 b	16.2 a	17.0 a	77.9 b	104.6 a	85.9 a	0.300 d
LSD (*p* < 0.05)	0.47	0.53	0.54	0.61	0.020	0.016	0.7	0.8	4.9	4.6	0.8	0.013

Means averaged across two water amounts; they differ at *p* < 0.05 when followed by different letter(s).

## Data Availability

The data used for construction of the adopted genomic selection models are freely available (a) in Data set S1 of [29], for adaptation of lines to soil types; (b) in Online Resource 3 of [39], for adaptation of lines to soil types; and (c) in Data set S1 of [25], for adaptation of lines to drought. The experimental data for the validation experiment are provided as a supplementary data set.

## Supplementary Materials

Supporting information can be downloaded at: https://www.mdpi.com/article/10.3390/ijms27094057/s1.

## Abbreviations

The following abbreviations are used in this manuscript:

ANOVA	Analysis of variance
GBS	Genotyping-by-sequencing
GEI	Genotype × environment interaction
GS	Genomic selection
LSD	Least Significant Difference
SNP	Single-Nucleotide Polymorphism
rrBLUP	Ridge regression best linear unbiased prediction
WGBLUP	Weighted G-BLUP
