# Supplementary material for: White Lupin Genomic Selection for Adaptation to Drought or Moderately Calcareous Soil: A Proof-of-Concept Study
Source: Int J Mol Sci. 2026 Apr 30;27(9):4057. doi: 10.3390/ijms27094057 (PMC13163514; doi:10.3390/ijms27094057)
Supplement: Supplementary file 1 [file ijms-27-04057-s001.zip › Supplementary Material.pdf]

Supplementary Material

Table S1. *F* test significance for seven traits in the analysis of variance of 12 white lupin genotypes grown in four managed environments issued by the factorial combination of two water amounts (drought stress; moisture-favorable) by two soil types (sandy-loam, non-calcareous; silty-clay, moderately calcareous).

Source of variation	Degrees of freedom	Grain yield	Straw biomass	Harvest index	Flowering time	Maturity time	Plant height	Seed weight
Genotype (G)	11	**	**	**	**	**	*	**
Environment (E)	3	**	**	**	*	**	**	NS
- Water amount (W)	1	**	**	**	**	**	**	NS
- Soil type (S)	1	**	**	**	NS	**	**	NS
- W × S	1	NS	*	**	NS	**	*	NS
Block in S × W	12	–	–	–	–	–	–	–
G × E	33	**	**	**	*	**	**	**
- G × W	11	**	**	**	NS	NS	NS	**
- G × S	11	**	**	**	**	**	**	**
- G × W × S	11	**	**	**	NS	**	NS	NS
Pooled error	132	–	–	–	–	–	–	–

NS, not significant; *, significant at $p < 0.05$; **, significant at $p < 0.01$.

Table S2. *F* test significance for seven traits in the analysis of variance performed separately for three white lupin sets of four genotypes each relative to breeding lines (BL) or landrace genotypes (LG) grown in four managed environments issued by the factorial combination of two water amounts (drought stress; moisture-favorable) by two soil types (sandy-loam, non-calcareous; silty-clay, moderately calcareous). Results for sources of variation relative to genotype (G) and its interaction with water amount (W) or soil type (S) factors.

Genotype set	Trait	G	G x W	G x S	G x W x S
BL - Adaptation to drought	Grain yield	**	**	**	*
BL - Adaptation to drought	Straw yield	**	**	*	NS
BL - Adaptation to drought	Harvest index	**	**	NS	NS
BL - Adaptation to drought	Flowering onset	**	NS	*	NS
BL - Adaptation to drought	Maturity time	**	**	**	NS
BL - Adaptation to drought	Plant height	**	NS	NS	NS
BL - Adaptation to drought	Seed weight	**	NS	**	NS
LG - Adaptation to drought	Grain yield	**	NS	NS	NS
LG - Adaptation to drought	Straw yield	**	*	*	NS
LG - Adaptation to drought	Harvest index	**	**	**	NS
LG - Adaptation to drought	Flowering onset	**	NS	NS	NS
LG - Adaptation to drought	Maturity time	**	NS	**	NS
LG - Adaptation to drought	Plant height	**	NS	*	NS
LG - Adaptation to drought	Seed weight	**	NS	**	NS
BL - Adaptation to moderately calcareous soil	Grain yield	**	NS	**	**
BL - Adaptation to moderately calcareous soil	Straw yield	**	NS	*	*
BL - Adaptation to moderately calcareous soil	Harvest index	**	*	**	**
BL - Adaptation to moderately calcareous soil	Flowering onset	**	NS	*	NS
BL - Adaptation to moderately calcareous soil	Maturity time	**	NS	NS	NS
BL - Adaptation to moderately calcareous soil	Plant height	*	NS	*	NS
BL - Adaptation to moderately calcareous soil	Seed weight	**	**	NS	NS

NS, not significant; *, $p < 0.05$; **, $p < 0.01$.
